# Efficacy of fluorides and CPP-ACP vs fluorides monotherapy on early caries lesions: A systematic review and meta-analysis

**DOI:** 10.1371/journal.pone.0196660

**Published:** 2018-04-30

**Authors:** Siying Tao, Yan Zhu, He Yuan, Sibei Tao, Yiming Cheng, Jiyao Li, Libang He

**Affiliations:** 1 State Key Laboratory of Oral Diseases, National Clinical Research Center for Oral Diseases, Department of Cariology and Endodontics, West China Hospital of Stomatology, Sichuan University, Chengdu, China; 2 Renal Division, Department of Internal Medicine, West China Hospital of Sichuan University, Chengdu, China; RWTH Aachen University, GERMANY

## Abstract

The study aimed to evaluate the efficacy of the combination of CPP-ACP and fluorides compared with fluorides monotherapy on patients with early caries lesions. The Medline, Embase and Cochrane databases up to August 2017 were scanned, with no restrictions. Studies satisfied the guideline of randomised controlled trials (RCTs), the patients with early caries lesions and data considering the efficacy of fluorides and CPP-ACP versus fluorides alone were selected. There was no language restriction during the literature search process, however, only papers in English or Chinese were included during the selection process. Outcome variables include laser fluorescence, quantitative light-induced fluorescence, lesion area and visual inspection scores. Mean differences were calculated during the data extraction process. Ten studies including 559 patients were selected in the meta-analysis. Fluorides combined with CPP-ACP achieved the same efficacy for early caries lesions on smooth surfaces compared with fluorides monotherapy (mean difference: -13.90, 95% confidence interval: [-39.25, 11.46], *P* = 0.28), and the combination treatment showed significantly better efficacy than fluorides monotherapy for occlusal early caries lesions (mean difference: -21.02, 95% confidence interval: [-27.94, -14.10], *P*<0.01). However, further well-designed studies are still needed.

## Introduction

Dental caries is one of the most prevalent chronic diseases of humans all over the world [1]. Its consequences, such as oral pain and tooth loss pose uncomfortable and loss-of-function problems especially in developing countries [2,3]. Risk factors involved in the caries process were the bacteria in biofilms, dietary sugars, host tooth condition and time. The pathological mechanism of early caries has been well recognised. Acidic by-products, generated by bacterial fermentation of dietary carbohydrates in the biofilm dissolve minerals causing the demineralisation of enamel [1,4–7]. Caries should be halted and reversed at the early stage to prevent the development of tooth decay. With the changing concept of caries management, minimal intervention dentistry, which attempts to preserve the tooth structure as much as possible, is intended to change routine clinical practice [8,9]. Therefore, remineralisation is indispensable for reversing the early carious lesions.

Fluoride therapy has been the cornerstone of the non-invasive treatments for early carious lesions [5]. Fluoride can facilitate calcium and phosphate diffusion into the demineralised lesions to remineralise the crystalline structures. The rebuilt crystalline structures, composed of fluoridated hydroxyapatite and fluorapatite, are much more resistant to acid attack than the original ones. Furthermore, fluoride can also affect cariogenic bacterial metabolism through several complex mechanisms [10,11]. Various types of fluoride therapy were applied with different recommended concentrations, frequency of use and dosage.

Casein phosphopeptide–amorphous calcium phosphate (CPP-ACP), a nanocomplex derived from milk, can stabilise higher concentrations of calcium and phosphate in an amorphous state to enhance remineralisation[12,13]. In recent years, studies have shown its potential to remineralise the early caries lesions [12,14–16] and also its anticariogenic characteristics in laboratory, animal, and human *in situ* experiments [17–20]. Nevertheless, there was currently some controversy regarding the efficacy of CPP-ACP and fluorides in the prevention of caries. According to the evidence revealed in two systematic reviews [21,22], CPP-ACP alone was not considered as “the best clinical practice” but the combination of CPP-ACP and fluorides could achieve better effects than CPP-ACP alone. These two systematic reviews focused on the caries-prevention effects of fluorides. Systematic reviews about the treatment effects of fluorides or fluorides with other treatment combinations have not been found, which have high clinical guiding significance and are still needed. Other trials concluded that the combination of CPP-ACP and fluorides is not superior to fluorides monotherapy [23–26]. Theses persistent controversy makes it difficult for dentists to choose the proper clinical treatments.

Thus, the aim of this study is to address the efficacy of the combination of CPP-ACP and fluorides versus fluorides monotherapy on patients with early caries lesions by performing a comprehensive systematic review and meta-analysis. The study protocols are shown in S1 Appendix.

## Materials and methods

This meta-analysis was performed according to the Preferred Reporting Items of Systematic Reviews and Meta-Analyses (PRISMA) items [27]. The PRISMA is a concise checklist consisting of 27 items deemed essential for reporting a clear and complete systematic review. The PRISMA checklist of this meta-analysis can be seen in S2 Appendix.

### Literature search

To recognise all relevant studies on the efficacy of fluorides with CPP-ACP or fluorides alone on early caries lesions, we searched Medline via PubMed (January 1990 until August 2017), EMBASE (January 1990 until August 2017), and Cochrane Central Register of Controlled Trials (January 1990 until August 2017). All relevant studies were reviewed. Both MeSH heading words and free text words were included during literature search. The search equations used in each database are elaborated in S3 Appendix. In case of additional relevant papers, reference lists of all targeted studies were searched manually.

### Selection criteria

#### Types of studies

This meta-analysis included all the randomised controlled trials (RCTs), which were parallel design clinical human trials comparing the efficacy of fluorides and CPP-ACP with fluorides alone. Reports without clinical data based on the effectiveness of treatment were excluded.

#### Types of participants

Studies involving subjects having early caries lesions in their permanent teeth were selected in our meta-analysis. All participants lived in an area where the water supply was non-fluoridated. Subjects with systemic diseases or proven/suspected milk protein allergy were excluded.

#### Types of intervention

Fluorides with CPP-ACP versus fluorides alone were included. The fluorides could include any kind of products containing fluorides, such as fluoride toothpastes, mouth rinses or varnishes. The CPP-ACP could include any kind of products containing CPP-ACP, such as MI Paste or Tooth Mousse, which are trademarks of products containing CPP-ACP.

### Outcome measures

Included efficacy outcomes were measured instrumentally and/or visually. (1) Laser fluorescence (LF) was used to assess the degree of early caries lesions. (2) Quantitative light-induced fluorescence (QLF) was used to assess the degree of early caries regions compared to the surrounding healthy tooth structure. (3) The value of total lesion area divided by total surface area of teeth tested was used to evaluate the area of early caries lesions. (4) Visual inspection scores were used to evaluate visual improvement after treatment.

Measurements (1) and (2) were evaluated instrumentally while measurement (3) and (4) was evaluated visually. Measurement (4) was not able to be included in the quantitative synthesis because the lesions were scored according to different criteria and values of standard deviation were not reported. Thus, results of measurement (4) were reviewed instead of a quantitative meta-analysis.

### Data extraction and quality assessment

For inclusion in this meta-analysis, two authors (S.T. and Y.Z.) inspected the titles and abstracts individually. Full-text papers were assessed when information available from titles and abstracts was not sufficient. A data extraction form was used to extract data in every single paper, which was conducted by two authors independently. The extracted data were made up of three components: study characteristics, patient characteristics and treatment outcomes. The study characteristics included publication date, sample size, follow-up period and type of intervention (type of combination, frequency and duration of therapy). The patient characteristics included demographic factors (sex and age) and clinical factors (location of lesions, mean value of laser fluorescence) at baseline. Whenever consensus could not be reached between the two reviewers, a third reviewer (H.Y.) made the decision.

We used the Cochrane Collaboration methodology [28] for assessing the risk of bias of every single study included. The domains evaluated included random sequence generation, allocation concealment, blinding of assessment, incomplete outcome data, selective reporting and other possible sources of bias.

### Statistical analysis

Mean difference (MD) for continuous data was calculated with a 95% confidence interval (CI) for generalising effectiveness of treatment in every single report. We used the random effects models to combine the studies due to the clinical and methodological heterogeneity existing in the studies [29]. Heterogeneity of studies was evaluated using the I^2^ statistical index. I^2^ > 50% indicated a high heterogeneity [30]. RevMan statistical software version 5.3 was used to conduct the analyses. *P*<0.05 was considered statistically significant, but when performing the test of heterogeneity, *P*<0.1 was considered statistically significant [28].

### Sensitivity analysis

To evaluate the robustness of the meta-analysis results, two sensitivity analyses were conducted: (1) high-quality studies versus low-quality studies; (2) studies with small sample size versus studies with large sample size.

## Results

### Search results and study characteristics

**Fig 1:**
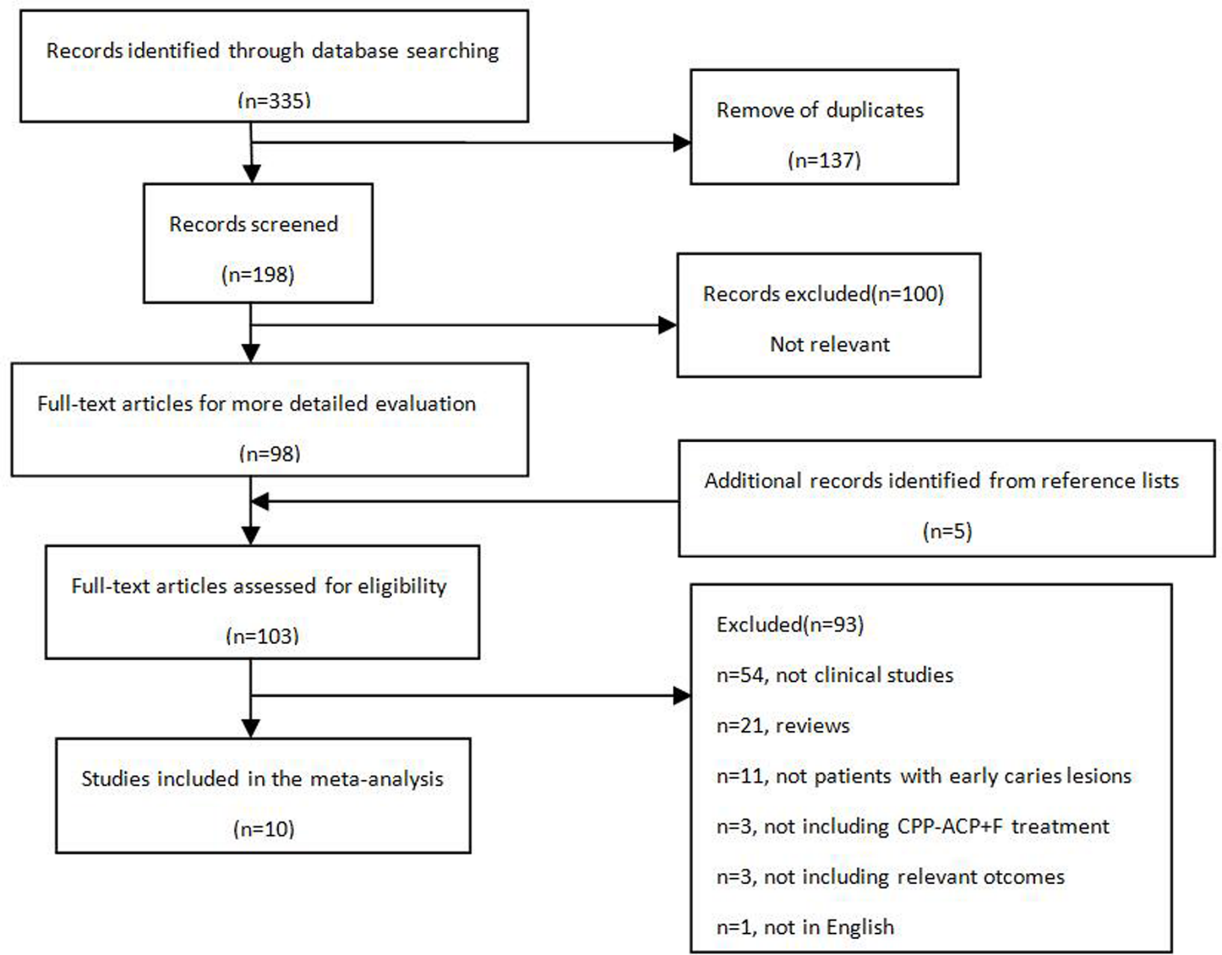
Flow diagram of the literature search process.

Through the literature search, 335 studies were identified, including 137 duplicates. Among the remaining 198 studies, 10 studies were eventually selected for this meta-analysis. A flow diagram of the studies which were identified, screened, assessed for eligibility, excluded and included in this analysis is displayed in Fig 1. The main characteristics of the included ten studies were summarised in Table 1, and the risk of bias was shown in Table 2. One [23] of the ten included had high risk of bias, three [24,26,31] had low risk of bias, while the other six [25,32–36] studies had unclear risk of bias.

**Table 1:** Main characteristics of included trials.

First author (Year)	Sample (Mean age)	Follow up (weeks)	Male (n,%)	Location of lesions	CPP-ACP+Fluorides Group			Fluorides Group		
					Type of combination	Frequency	Mean LF	Type of combination	Frequency	Mean LF
Llena 2015 [23]	80(n.r)	12	n.r	os	TM+Ft	qd+n.r	6.29	NaF v+Ft	monthly+n.r	6.40
			ss	TM+Ft	qd+n.r	3.61	NaF v+Ft	monthly+n.r	4.45	
Aykut 2014 [32]	56(13)	12	n.r	ss	TM+Ft	bid+bid	14.31	Ft	bid	14.14
Fredrick 2013 [33]	45(n.r)	4	26,57.8%	os	TMP	bid	18.2	NaF mr	bid	18.6
Huang 2013 [24]	115(14.4)	8	56,48.7%	ss	MIPP+Ft	bid+n.r	n.r	NaF v+Ft	once+n.r	n.r
Bröchner 2011 [25]	50(15.2)	4	n.r	ss	TM+Ft	qd+qd	n.r	Ft	bid	n.r
Beerens 2010 [26]	54(15.5)	12	23,42.6%	ss	MIPP+Ft	qd+bid	n.r	Ft	qd+bid	n.r
Akin 2012 [34]	80(14.5)	24	n.r	ss	TM+Ft	bid+bid	n.r	NaF mr+Ft	bid+bid	n.r
Andersson 2007 [36]	26(14.6)	24	13,50%	ss	To+Ft	bid(12w)+bid(12w)	7.4	NaFmr+Ft	qd+bid	9.4
Altenburger2010 [35]	32(25.5)	3	16,50%	os	TM+Ft	qd+bid	16.66	Ft	bid	16.87
Guclu 2016 [31]	21(n.r)	12	13, 61.9%	ss	TM+ NaF v	5 times in 12w	16.5	NaF v	5 times in 12w	16.9

Explanations: “n.r” means “not reported”, “qd” means “once daily”, “bid” means “twice daily”, “w” means “weeks”, “LF” means “the value of laser fluorescence”, “os” means “occlusal surfaces”, “ss” means “smooth surfaces”, “Ft” means “Fluoride toothpaste”, “v” means “varnish”, “mr” means “mouth rinse”. “MIPP” means “MI Paste Plus”, “TM” means “Tooth Mousse”, “TMP” means “Tooth Mousse Plus”, “To” means “Topacal”. These are trademarks of products. MI Paste Plus contains 10% CPP-ACP and 900ppm fluoride, Tooth Mousse Plus contains 10% CPP-ACP and 0.2% NaF, Tooth Mousse contains 10% CPP-ACP. Topacal contains CPP-ACP without fluoride. Fluoride toothpaste contains 1000-1450ppm fluoride. NaF varnish contains 5% sodium fluoride.

**Table 2:** Ascertainment of the risk of bias in the included studies.

Study	Random sequence generation	Allocation concealment	Blinding of assessment	Incomplete outcome data	Selective reporting		Other sources of bias	Risk of bias	
Llena 2015 [23]	?	?	-	+	-	-			High
Aykut 2014 [32]	?	-	-	?	-	-			Unclear
Fredrick 2013 [33]	?	?	-	-	-	-			Unclear
Huang 2013 [24]	-	-	-	-	-	-			Low
Bröchner 2011 [25]	?	?	?	-	-	-			Unclear
Beerens 2010 [26]	-	-	-	-	-	-			Low
Akin 2012 [34]	?	?	-	-	-	-			Unclear
Andersson 2007 [36]	?	?	-	-	-	-			Unclear
Altenburger 2010 [35]	?	?	-	-	-	-			Unclear
Guclu 2016 [31]	-	-	-	-	-	-			Low

Explanations: “+” means this item would increase the risk of bias, “-” means this item would decrease the risk of bias, “?” means this item was not clearly reported in the study so that it couldn’t be assessed accurately.

### Effectiveness of treatment

#### Laser fluorescence (LF)

Six [23,31,32,33,35,36] of the ten studies assessed efficacy using the values of LF. Llena *et a*l [23] included early caries lesions on both occlusal surfaces and smooth surfaces, and reported results for occlusal caries and smooth surface lesions respectively. Therefore, LF values in this study were extracted separately according to the location of lesions, which were expressed as two studies: “Llena 2015 os” and “Llena 2015 ss” in the forest plot respectively. “Os” means lesions on occlusal surfaces and “ss” means lesions on smooth surfaces. That’s the reason why there are seven studies shown in the forest plot. An LF related item was integrated in each study to avoid individual bias and heterogeneity between studies. This LF related item represented the change rate of LF values. Subgroup analysis was conducted according to different location of lesions.

**Fig 2:**
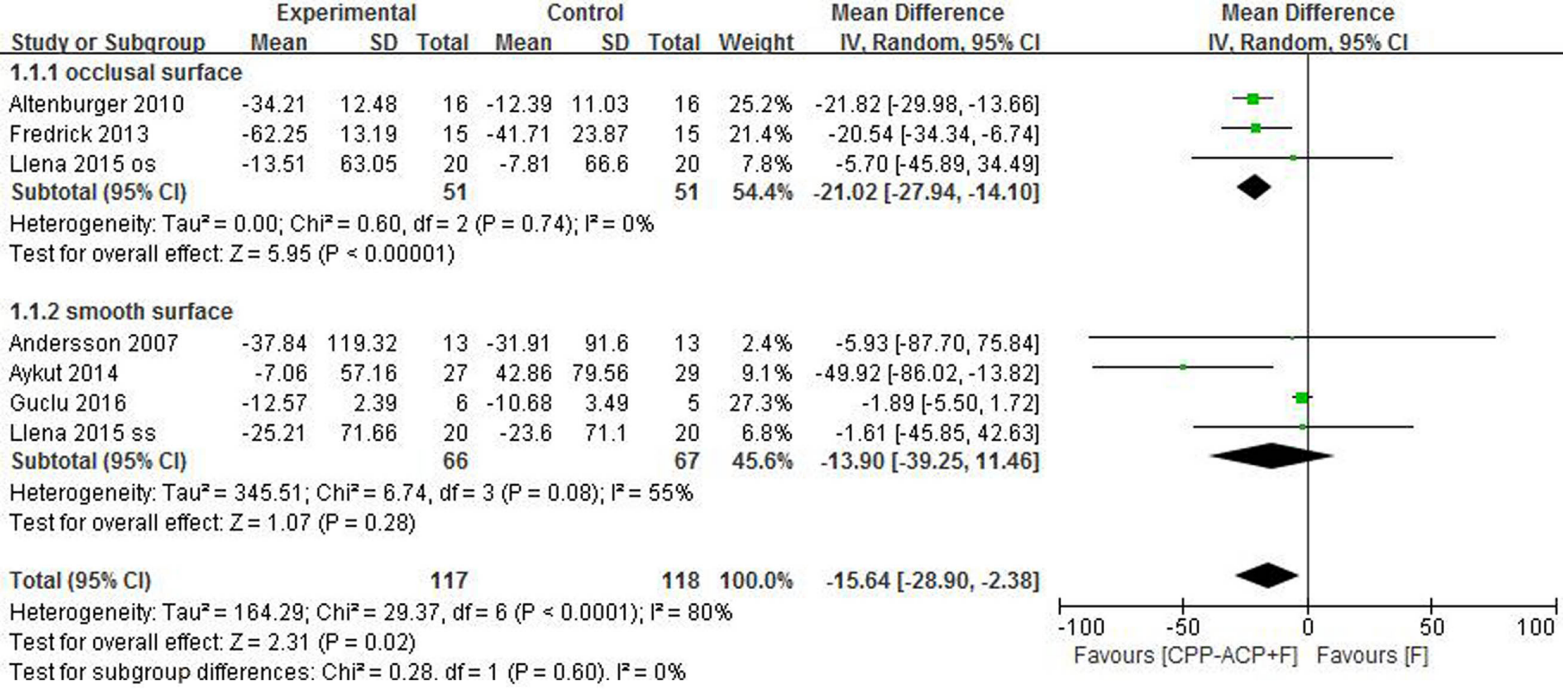
Efficacy in CPP-ACP and fluorides vs fluorides alone using LF value.

A subgroup analysis of three studies [23,33,35] including lesions on occlusal surfaces showed that the use of CPP-ACP together with fluorides produced better efficacy (MD = -21.02, 95% CI: [-27.94, -14.10], *P*<0.00001). Heterogeneity was not observed between the studies(P = 0.74, I^2^ = 0%). In the four studies [23,31,32,36] including lesions on smooth surfaces, the subgroup analysis showed no significant difference between using fluorides together with CPP-ACP and using fluorides alone (MD = -13.90, 95% CI: [-39.25, 11.46], *P* = 0.28) (Fig 2).

#### Quantitative light-induced fluorescence (QLF)

**Fig 3:**
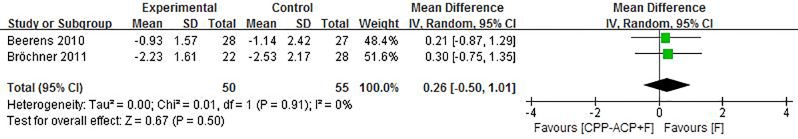
Efficacy in CPP-ACP and fluorides vs fluorides alone using QLF value.

Two studies [25,26] assessed efficacy using the values of QLF. Both studies included early caries lesions on smooth surfaces. Heterogeneity was not detected between the studies (*P* = 0.91, I^2^ = 0%). Meta-analysis showed no significant difference between using fluorides together with CPP-ACP and using fluorides alone (MD = 0.26, 95% CI: [-0.50, 1.01], *P* = 0.50) (Fig 3).

#### Total lesion area divided by total surface area of teeth tested

**Fig 4:**
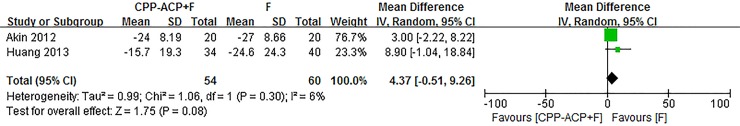
Efficacy in CPP-ACP and fluorides vs fluorides alone using the value of total lesion area divided by total surface area of teeth tested.

Two [24,34] studies assessed efficacy using the value of total lesion area divided by total surface area of teeth tested. Both studies included early caries lesions on smooth surfaces. Heterogeneity was not significant between the studies (*P* = 0.30, I^2^ = 6%). Meta-analysis did not demonstrate a significant difference between these two kinds of treatment when comparing total lesion area divided by total surface area of teeth tested, which was assessed by visual measurement (MD = 4.37, 95% CI: [-0.51, 9.26], *P* = 0.08) (Fig 4).

#### Visual inspection scores

Two trials [25,36] evaluated visual inspection scores before and after these two kinds of treatments. Bröchner *et al* [25] demonstrated that fluorides combined with CPP-ACP did not show significant lower scores than fluorides monotherapy using criteria according to Gorelic *et al* [37] (mean scores before/after treatment in fluorides with CPP-ACP group: 2.159/1.675, mean scores before/after treatment in fluorides monotherapy group: 2.123/1.591). Andersson *et al* [36] concluded that more lesions became invisible in the fluorides with CPP-ACP group than the fluorides monotherapy group using criteria according to Andersson *et al* [38] (mean scores before/after treatment in fluorides with CPP-ACP group: 2.87/2.08, mean scores before/after treatment in fluorides monotherapy group: 2.87/1.44). Conclusions in two trials were different may due to different criteria. Criteria in future studies should be standardized to make quantitative synthesis possible. Meta-analysis could not be performed comparing visual inspection scores of lesions in two kinds of treatment because data available in both studies was limited.

### Sensitivity analysis

We conducted sensitivity analysis for trials with large sample size and low risk of bias. Eight [23,24,25,26,32,33,34,35] of the ten included studies were with large sample size, and three [24,26,31] of the ten included studies were with low risk of bias. When eliminating studies with small sample size or high risk of bias, all results were consistent to the meta-analysis results using all ten studies.

## Discussion

Non-invasive therapy by remineralisation to treat early caries lesions has been proved to be of significant benefit in clinical practice [39,40,41]. Treatment with fluorides has been shown to enhance the rate of remineralisation and has been widely used to increase the remineralisation of early carious lesions. Fluoride-containing products are more and more widely available, including toothpastes, gels, mouth rinses and varnishes. Casein phosphopeptides (CPP) have the ability to stabilise the amorphous calcium phosphate (ACP). The action of the CPP-ACP complex ranges from buffering pH, preventing demineralisation to enhancing remineralisation. In many recent studies which focused on the treatment of early caries lesions, CPP-ACP has shown promising outcomes as an adjunctive treatment to fluorides [15]. Moreover, CPP-ACP is a unique protein derived from milk, and therefore has a high safety level. A number of *in vitro*, *in situ* and *in vivo* experiments have indicated the possible anticariogenic ability of CPP-ACP by its remineralising effects [18,32–35,42–45]. However, studies [31,36] demonstrating that fluorides combined with CPP-ACP achieved no clinical advantage over fluorides alone, which might question the anticariogenic ability of CPP-ACP. Our study concluded that fluorides combined with CPP-ACP treatment produces significantly better efficacy for occlusal early caries lesions. For lesions on smooth surfaces, fluorides monotherapy may achieve the same effectiveness. Other non-invasive treatments for early caries lesions were also reported. Fluorides with tricalcium phosphate did not show any significant advantage for the prevention of enamel demineralisation [46,47]. The combination of CPP-ACP and photo-activated disinfection might be effective as a treatment for stabilising root surface caries, as described in a case report [48].

Some limitations in this meta-analysis should be addressed. This review included studies recruiting patients with early caries lesions and compared two medical strategies. Therefore, this evidence is not applicable to the population without caries lesions. The preventive effect on dental caries using CPP-ACP with fluorides or fluorides alone cannot be ascertained through this meta-analysis. The efficacy of other kinds of treatment for early caries lesions (such as microabrasion treatment [34]) was not discussed. The limited number of studies included resulted in tiny subgroups, which suggests that the evidence is incomplete and is not generalisable. A judgment of the quality of evidence in the meta-analysis is offered by Table 1 (main characteristics of included studies) and Table 2 (risk of bias for each study). This review selected ten studies evaluating two kinds of treatment applied in 559 patients with early caries lesions. Robust conclusions are inferred due to this sample size. However, we were still limited by the data (both quantity and type) available to us, as were all other meta-analyses. To make meta-analysis possible, we adjusted for the different types of data using statistical processes. There is considerable risk, that by doing so, we might lower the quality of the evidence. There could be a bias in the outcome since we analyzed different products and different treatment frequency together. For example, fluorides with CPP-ACP group included both “Tooth Mousse+fluoride toothpaste” and “MI Paste Plus+fluoride toothpaste”. MI Paste Plus contains CPP-ACP and NaF, while Tooth Mousse contains CPP-ACP without NaF, which has been expounded in Table 1 explanations. Different locations of lesions, different kinds of products, different treatment frequency could all induce heterogeneity within studies when comparing outcomes. We conducted subgroup analysis according to different locations of lesions, which reduced I^2^ from 80% to 0% and 55%, respectively (Fig 2). This indicated that lesion location was the main factor inducing heterogeneity, and subgroup analysis made the outcomes have comparability. During the selection process, rigid inclusion criteria minimised the potential of bias. Reviewers evaluated papers independently and disagreement was solved either through discussion or by consulting a third reviewer if needed.

Laser fluorescence (LF) and quantitative light-induced fluorescence (QLF) are new appropriate techniques used for diagnosing caries located both in occlusal and smooth surfaces [49]. Pits and fissures on occlusal surfaces have thinner enamel layer than smooth surfaces. Dental plaque are more likely to accumulate at pits and fissures because of their special anatomic structure. Therefore, occlusal surfaces are more susceptible to caries than smooth surfaces. It is much more difficult to clean pits and fissures than smooth surfaces using toothbrush, so pit and fissure caries progresses more rapidly than smooth surface lesions. It is also more difficult for medication in the form of paste, gel, varnish or mouth rinse to act on pit and fissure lesions compared with smooth surface lesions because of anatomic structure. Although caries experience has dropped over the last 30 years majorly because of the increased access to fluorides, caries on occlusal surfaces decreases by much smaller proportion compared to smooth surfaces [50–52]. These facts may confirm our results that the combination of CPP-ACP and fluorides is superior to fluorides monotherapy for occlusal early caries lesions while fluorides alone seem enough for smooth surface lesions. We cannot draw a definite conclusion at present because of the limited evidence. Besides, LF is not as accurate as QLF in detecting mineral changes, which has been shown by numerous studies [53–56]. LF and QLF are instrumental evaluation, while lesion area is visual evaluation. Thus, LF and QLF would be more appropriate outcomes than lesion area in our study. We could not draw a definite conclusion now in respect to visual inspection scores of the two treatments due to limitations in the original papers. Future more studies are needed to draw a conclusion about additional visual improvement function of CPP-ACP. Most of the included studies in our meta-analysis used LF values to compare the efficacy of two kinds of treatment, therefore, future studies using QLF as instrumental measurement to assess the effectiveness are needed to confirm our results. Studies included in this meta-analysis had follow-up time ranging from 3 to 24 weeks, which were relatively short-term. A previous study evaluated the effect of 3 months application of CPP-ACP with a follow up of 12 months, and CPP-ACP still showed effective [57], which indicated the promising effects of CPP-ACP. However, future long-term studies with longer than 5-year follow-up time are still needed, following other long-term studies in endodontic and restorative dentistry [58, 59].

When treating patients with early caries lesions, dentists are recommended to make treatment plans according to the location of lesions. For occlusal lesions, it is better to use CPP-ACP together with fluorides. For lesions on smooth surfaces, there is no significant difference between using CPP-ACP with fluorides and using fluorides alone, however, the combination treatment may indicate new ideas for reducing the use of fluorides especially when treating children. Further studies in the future are still needed to explore the advantages of CPP-ACP in clinical treatment.

## Conclusions

CPP-ACP seems to have a good efficacy for treatmrent of early caries lesions on occlusal tooth surfaces within the limitation of our study. The combination of fluorides and CPP-ACP may achieve the same effectiveness as fluorides monotherapy when the lesions are on smooth surfaces. For occlusal lesions, CPP-ACP and fluorides combination treatment may even improve the efficacy compared with fluorides monotherapy. However, more large-sample rigorous studies in the future are needed to confirm the advantages of CPP-ACP in treating early caries lesions.

## Supporting information

S1 Appendix Study protocols. (DOCX)

S2 Appendix PRISMA checklist. (DOC)

S3 Appendix Search equations. (DOCX)
