# Supplementary material for: Efficacy of fluorides and CPP-ACP vs fluorides monotherapy on early caries lesions: A systematic review and meta-analysis
Source: PLoS One. 2018 Apr 30;13(4):e0196660. doi: 10.1371/journal.pone.0196660 (PMC5927405; doi:10.1371/journal.pone.0196660)
Supplement: S1 Appendix — (DOCX) [file pone.0196660.s001.docx]

**Protocols**

**Efficacy of Fluorides and CPP-ACP vs Fluorides Monotherapy on Early Caries Lesions: a Systematic Review and Meta-Analysis**

Siying Tao^1^, Yan Zhu^1^, He Yuan^1^, Sibei Tao ^2^, Yiming Cheng^2^, Jiyao Li^1^, Libang He^1*^

**Author Information**

^1^ State Key Laboratory of Oral Diseases, National Clinical Research Center for Oral Diseases, Department of Cariology and Endodontics, West China Hospital of Stomatology, Sichuan University, Chengdu 610041, China.

^2^ Renal Division, Department of Internal Medicine, West China Hospital of Sichuan University, Chengdu, 610041, Sichuan, China.

**Background**

Dental caries is one of the most prevalent chronic diseases of humans all over the world^1^. Its consequences, such as oral pain and tooth loss pose uncomfortable and loss-of-function problems especially in developing countries^2,3^. Risk factors involved in the caries process were the bacteria in biofilms, dietary sugars, host tooth condition and time. The pathological mechanism of early caries has been well recognised. Acidic by-products, generated by bacterial fermentation of dietary carbohydrates in the biofilm dissolve minerals causing the demineralisation of enamel^1,4-7^. Caries should be halted and reversed at the early stage to prevent the development of tooth decay. With the changing concept of caries management, minimal intervention dentistry, which attempts to preserve the tooth structure as much as possible, is intended to change routine clinical practice^8,9^. Therefore, remineralisation is indispensable for reversing the early carious lesions.

Fluoride therapy has been the cornerstone of the non-invasive treatments for early carious lesions^5^. Fluoride can facilitate calcium and phosphate diffusion into the demineralised lesions to remineralise the crystalline structures. The rebuilt crystalline structures, composed of fluoridated hydroxyapatite and fluorapatite, are much more resistant to acid attack than the original ones. Furthermore, fluoride can also affect cariogenic bacterial metabolism through several complex mechanisms^10,11^. Various types of fluoride therapy were applied with different recommended concentrations, frequency of use and dosage.

Casein phosphopeptide–amorphous calcium phosphate (CPP-ACP), a nanocomplex derived from milk, can stabilise higher concentrations of calcium and phosphate in an amorphous state to enhance remineralisation^12,13^. In recent years, studies have shown its potential to remineralise the early caries lesions^12,14-16^ and also its anticariogenic characteristics in laboratory, animal, and human in situ experiments^17-20^. Nevertheless, there was currently some controversy regarding the efficacy of CPP-ACP and fluorides in the prevention of caries. According to the evidence revealed in two systematic reviews^21,22^, CPP-ACP alone was not considered as “the best clinical practice” but the combination of CPP-ACP and fluorides could achieve better effects. Other trials concluded that the combination of CPP-ACP and fluorides is not superior to fluorides monotherapy^23-26^. The persistent controversy makes it difficult for dentists to choose the proper clinical treatments.

Thus, the aim of this study is to address the efficacy of the combination of CPP-ACP and fluorides versus fluorides monotherapy on patients with early caries lesions by performing a comprehensive systematic review and meta-analysis.

**Objectives**

To evaluate the efficacy of the combination of CPP-ACP and fluorides compared with fluorides monotherapy on patients with early caries lesions.

**Methods**

**Criteria for considering studies for this review**

***Types of studies***

We will review all randomized controlled trials (RCTs), parallel design, that compare the the efficacy of fluorides and CPP-ACP with fluorides alone in patients with early caries lesions. Following the steps listed in the Preferred Reporting Items for Systematic Reviews and Meta-Analyses (PRISMA)^27^. We will identify appropriate studies, extract data, conduct meta-analysis and report review results.

***Types of participants***

Our meta-analysis will include subjects having early caries lesions in their permanent teeth.

***Types of interventions***

We will include RCTs comparing two combination therapies: Fluorides with CPP-ACP and fluorides alone. The fluorides include any kind of products containing fluorides, such as fluoride toothpastes, mouth rinses or varnishes. The CPP-ACP can include any kind of products containing CPP-ACP, such as MI Paste or Tooth Mousse, which are trademarks of products containing CPP-ACP.

***Types of outcome measures***

(1) Laser fluorescence (LF). (2) Quantitative light-induced fluorescence (QLF). (3) The value of total lesion area divided by total surface area of teeth tested.

**Search methods for identification of studies**

We will retrieve relevant articles by searching Pubmed, Embase, Medline and the Cochrane databases up to 2017. Key words in the searches will include casein phosphopeptide amorphous calcium phosphate, CPP-ACP, casein derivate, milk derivate, fluoride, dental caries, enamel demineralisation, white spot lesion, remineralisation.

**Data collection and analysis**

***Selection of studies***

The search strategy and selection of this meta-analysis were discussed by all reviewers and finally formed to be a written file. Two authors of the paper (Siying Tao and Yan Zhu) will independently conduct the literature search and data extraction. Upon comparison of their results, if consensus will not be reached through discussion, a third reviewer served as the arbitrator will decide whether a particular publication meets inclusion criteria.

***Data extraction and management***

For the articles in the final list, two investigators (Siying Tao and Yan Zhu) will independently extract data, and discrepancies will be resolved by consensus. The extracted data will consist of three components: study characteristics, patient characteristics, and outcomes. The study characteristics will include publication date, sample size, follow-up period and type of intervention (type of combination, frequency and duration of therapy). The patient characteristics will demographic factors (sex and age) and clinical factors (location of lesions, mean value of laser fluorescence) at baseline.

***Assessment of risk of bias in included studies***

We will assess the Cochrane Collaboration methodology^28^. The criteria include generation of allocation sequence, concealment of allocation, masking of subjects, staff and outcome assessors, completeness of data, selectiveness of outcome reporting.

***Measures of treatment effect***

We will conduct estimates of association for all studies using inverse variance weighted random-effects analysis with 95% confidence intervals (CIs). We will use the random effects model to calculate the effect sizes because of known clinical and methodological heterogeneity of the studies^29^. Heterogeneity will be calculated using the I^2^ statistical index. This index specifically describes the percentage of total variation due to heterogeneity rather than chance, with I^2^ more than 50% representing a high heterogeneity^30^. We will conduct random-effect meta-analysis to obtain estimates of outcomes, and then present these outcomes mean differences (continuous outcome, including laser fluorescence (LF), quantitative light-induced fluorescence (QLF), the value of total lesion area divided by total surface area of teeth tested) with 95% CIs. Analyses will perform using RevMan statistical software version 5.3.

***Unit of analysis issues***

The unit of analysis will be patients with early caries lesions according to the intervention group to which they were randomly assigned.

***Dealing with missing data***

We will perform an intention-to-treat analysis whenever possible. Otherwise, we will use the data that are available to us.

For continuous outcomes, we will impute the standard deviation from P values according to guidance given in the Cochrane Handbook for Systematic Reviews of Interventions. If the data are likely to be normally distributed, we will use the median for meta-analysis when the mean is not available. If it is not possible to calculate the standard deviation from the P value or the confidence intervals, we will impute the standard deviation using the largest standard deviation in other trials for that outcome. This form of imputation may decrease the weight of the study for calculation of mean differences and may bias the effect estimate to no effect for calculation of standardized mean differences.

***Assessment of heterogeneity***

Heterogeneity will be calculated using the I^2^ statistical index. This index specifically describes the percentage of total variation due to heterogeneity rather than chance, with I^2^ more than 50% representing a high heterogeneity.

***Assessment of reporting biases***

Reporting bias will be formally assessed using funnel plots and the Egger test, a measure that assesses the asymmetry of the funnel plot.

***Data synthesis***

As recommended by the Cochrane Collaboration, the meta-analysis will be conducted using the Review Manager (RevMan) software.

***Subgroup analysis and investigation of heterogeneity***

If possible, subgroup analyses will be conducted to explore possible sources of heterogeneity related to the location of leisions.

***Sensitivity analysis***

The robustness of the results will be tested using two sensitivity analyses: studies that are high quality versus low quality, and studies with small sample size versus large sample size.

**Acknowledgments**

This work is supported by the National Natural Science Foundation of China [grant number 81400508(L.H.)]; Specialized Research Fund for the Doctoral Program of Higher Education of China [grant number 20130181120125 (L.H.)].

**Contributions of authors**

Libang He coordinated the review and organised the team. Siying Tao and Yan Zhu contributed to develop the protocol. Siying Tao and Yan Zhu will assess the studies，extract data and perform the data analysis and interpretation. Siying Tao will draft the review. Yiming Cheng and Sibei Tao will edit and format the review text and give support of statistical methodology. He Yuan, Jiyao Li and Libang He will revise the article.

**Declaration of interests**

All authors declare no competing interests.

***References***

1. Selwitz RH, Ismail AI, Pitts NB. Dental caries. *Lancet.* 2007;369(9555):51-59.

2. Kidd E, Giedrys-Leeper E, Simons D. Take two dentists: a tale of root caries. *Dental update.* 2000;27(5):222-230.

3. Health UDo, Services H. Oral health in America: a report of the Surgeon General. *Rockville, MD: US Department of Health and Human Services, National Institute of Dental and Craniofacial Research, National Institutes of Health.* 2000;63:74-94.

4. Scheie AA, Petersen FC. The biofilm concept: consequences for future prophylaxis of oral diseases? *Critical Reviews in Oral Biology & Medicine.* 2004;15(1):4-12.

5. Fejerskov O. Changing paradigms in concepts on dental caries: consequences for oral health care. *Caries research.* 2004;38(3):182-191.

6. Featherstone J. The continuum of dental caries—evidence for a dynamic disease process. *Journal of dental research.* 2004;83(suppl 1):C39-C42.

7. Featherstone JD. The science and practice of caries prevention. *The Journal of the American Dental Association.* 2000;131(7):887-899.

8. Frencken JE, Peters MC, Manton DJ, Leal SC, Gordan VV, Eden E. Minimal intervention dentistry for managing dental caries–a review. *International dental journal.* 2012;62(5):223-243.

9. Pitts N. Are we ready to move from operative to non-operative/preventive treatment of dental caries in clinical practice? *Caries research.* 2004;38(3):294-304.

10. Koo H. Strategies to enhance the biological effects of fluoride on dental biofilms. *Advances in dental research.* 2008;20(1):17-21.

11. Marquis RE. Antimicrobial actions of fluoride for oral bacteria. *Canadian journal of microbiology.* 1995;41(11):955-964.

12. Prestes L, Souza BM, Comar LP, Salomão PA, Rios D, Magalhães AC. In situ effect of chewing gum containing CPP–ACP on the mineral precipitation of eroded bovine enamel—A surface hardness analysis. *Journal of dentistry.* 2013;41(8):747-751.

13. Cochrane N, Reynolds E. Calcium phosphopeptides—mechanisms of action and evidence for clinical efficacy. *Advances in dental research.* 2012;24(2):41-47.

14. Zhou C, Zhang D, Bai Y, Li S. Casein phosphopeptide–amorphous calcium phosphate remineralization of primary teeth early enamel lesions. *Journal of dentistry.* 2014;42(1):21-29.

15. Reynolds E, Cai F, Cochrane N, et al. Fluoride and casein phosphopeptide-amorphous calcium phosphate. *Journal of dental research.* 2008;87(4):344-348.

16. Manton DJ, Walker GD, Cai F, Cochrane NJ, Shen P, Reynolds EC. Remineralization of enamel subsurface lesions in situ by the use of three commercially available sugar‐free gums. *International Journal of Paediatric Dentistry.* 2008;18(4):284-290.

17. Morgan M, Adams G, Bailey D, Tsao C, Fischman S, Reynolds E. The anticariogenic effect of sugar-free gum containing CPP-ACP nanocomplexes on approximal caries determined using digital bitewing radiography. *Caries research.* 2008;42(3):171-184.

18. Iijima Y, Cai F, Shen P, Walker G, Reynolds C, Reynolds E. Acid resistance of enamel subsurface lesions remineralized by a sugar-free chewing gum containing casein phosphopeptide-amorphous calcium phosphate. *Caries research.* 2004;38(6):551-556.

19. Reynolds E, Cai F, Shen P, Walker G. Retention in plaque and remineralization of enamel lesions by various forms of calcium in a mouthrinse or sugar-free chewing gum. *Journal of dental research.* 2003;82(3):206-211.

20. Shen P, Cai F, Nowicki A, Vincent J, Reynolds E. Remineralization of enamel subsurface lesions by sugar-free chewing gum containing casein phosphopeptide-amorphous calcium phosphate. *Journal of dental research.* 2001;80(12):2066-2070.

21. Benson PE, Parkin N, Millett DT, Dyer F, Vine S, Shah A. Fluorides for the prevention of white spots on teeth during fixed brace treatment. *The Cochrane database of systematic reviews.* 2004;3.

22. Twetman S, Axelsson S, Dahlgren H, et al. Caries‐preventive effect of fluoride toothpaste: a systematic review. *Acta odontologica Scandinavica.* 2003;61(6):347-355.

23. Llena C, Leyda A, Forner L. CPP-ACP and CPP-ACFP versus fluoride varnish in remineralisation of early caries lesions. A prospective study. *European journal of paediatric dentistry: official journal of European Academy of Paediatric Dentistry.* 2015;16(3):181-186.

24. Huang GJ, Roloff-Chiang B, Mills BE, et al. Effectiveness of MI Paste Plus and PreviDent fluoride varnish for treatment of white spot lesions: a randomized controlled trial. *American Journal of Orthodontics and Dentofacial Orthopedics.* 2013;143(1):31-41.

25. Bröchner A, Christensen C, Kristensen B, et al. Treatment of post-orthodontic white spot lesions with casein phosphopeptide-stabilised amorphous calcium phosphate. *Clinical oral investigations.* 2011;15(3):369-373.

26. Beerens M, Van Der Veen M, Van Beek H, Ten Cate J. Effects of casein phosphopeptide amorphous calcium fluoride phosphate paste on white spot lesions and dental plaque after orthodontic treatment: a 3‐month follow‐up. *European journal of oral sciences.* 2010;118(6):610-617.

27. Liberati A, Altman DG, Tetzlaff J, et al. The PRISMA statement for reporting systematic reviews and meta-analyses of studies that evaluate health care interventions: explanation and elaboration. *Annals of internal medicine.* 2009;151(4):W-65-W-94.

28. Green S. Cochrane handbook for systematic reviews of interventions version 5.1. 0 [updated March 2011]. *The Cochrane Collaboration.* 2011.

29. Higgins JP, Thompson SG, Deeks JJ, Altman DG. Measuring inconsistency in meta-analyses. *Bmj.* 2003;327(7414):557-560.

30. Huedo-Medina TB, Sánchez-Meca J, Marín-Martínez F, Botella J. Assessing heterogeneity in meta-analysis: Q statistic or I² index? *Psychological methods.* 2006;11(2):193.
