# Supplementary material for: Efficacy of fluorides and CPP-ACP vs fluorides monotherapy on early caries lesions: A systematic review and meta-analysis
Source: PLoS One. 2018 Apr 30;13(4):e0196660. doi: 10.1371/journal.pone.0196660 (PMC5927405; doi:10.1371/journal.pone.0196660)
Supplement: S3 Appendix — (DOCX) [file pone.0196660.s003.docx]

**Database: PubMed (January 1990 until August 2017)**

#1 "Fluorides"[Mesh]

#2 "Tin Fluorides"[Mesh]

#3 "Fluorides, Topical"[Mesh]

#4 "Sodium Fluorides"[Mesh]

#5 "Caseins"[Mesh]

#6 “casein phosphopeptide amorphous calcium phosphate” OR “CPP-ACP” OR “casein derivate” OR “milk derivate” OR “fluoride” OR “dental caries” OR “enamel demineralisation” OR “white spot lesion” OR “remineralisation”

#7 #1 OR #2 OR #3 OR #4 OR #6

#8 #7 AND #5

**Database: EMBASE (January 1990 until August 2017)**

#3 #1 AND #2

#2 exp fluoride/

#1 exp casein/

**Database: EBM Reviews - Cochrane Central Register of Controlled Trials (January 1990 until August 2017)**

1 exp Caseins/

2 exp Fluorides/

3 exp Fluorides, Topical/

4 exp Tin Fluorides/

5 2 or 3 or 4

6 1 and 5
